# Tricuspid Regurgitation (TR) after Implantation of a Cardiac Implantable Electronic Device (CIED)—One-Year Observation of Patients with or without Left Ventricular Dysfunction

**DOI:** 10.3390/jcdd10080353

**Published:** 2023-08-19

**Authors:** Karolina Chodór-Rozwadowska, Magdalena Sawicka, Stanisław Morawski, Zbigniew Kalarus, Tomasz Kukulski

**Affiliations:** 1 Doctoral School, Department of Cardiology, Congenital Heart Diseases and Electrotherapy, Silesian Center for Heart Diseases, Faculty of Medical Sciences in Zabrze, Medical University of Silesia, 40-055 Katowice, Poland; 2 Department of Cardiology, Congenital Heart Diseases and Electrotherapy, Medical University of Silesia, 40-055 Katowice, Poland; z.kalarus@sccs.pl; 3 Department of Cardiac, Vascular and Endovascular Surgery and Transplantology, Silesian Center for Heart Diseases, Maria Skłodowska—Curie 9 Street, 41-800 Zabrze, Poland; msawicka155@gmail.com; 4 Department of Cardiac Transplantation and Mechanical Circulatory Support, Silesian Center for Heart Diseases, Maria Skłodowska—Curie 9 Street, 41-800 Zabrze, Poland; 5 Department of Cardiology, Silesian Centre for Heart Diseases, 41-800 Zabrze, Poland; morawski.stan@gmail.com; 6 2nd Department of Cardiology, Medical University of Silesia, Katowice Poland, Spec. Hospital, 41-808 Zabrze, Poland; t.kukulski@sccs.pl

**Keywords:** tricuspid regurgitation, cardiac implantable electronic device, heart failure

## Abstract

The frequency of tricuspid regurgitation (TR) progression after cardiac implantable electronic devices (CIEDs) implantation varies from 7.2% to 44.7%. TR is associated with increased mortality and hospitalizations due to heart failure (HF) decompensation. The aim of this study was to assess the rate of early TR progression after CIED implantation and the frequency of HF decompensation and mortality. The 101 patients, who received a CIED between March 2020 and October 2021, before the procedure were divided into two groups–one with left ventricle ejection fraction (LVEF) ≥ 40% (*n* = 60) and one with LVEF < 40% (*n* = 41). Lead-related tricuspid regurgitation (LRTR) was defined as an increase of TR by at least one grade. The follow-up period was similar between both groups and was on average 13 (12–16) months. In the whole study group, TR progression by one grade was 34.6% and by two or more grades 15.8%. The significant changes in the dynamic of TR degree were as follows before and after implantation: none/trivial TR in group 1 (61.7% vs. 28.3%, *p* = 0.01) and severe/massive TR in group 2 (0.0% vs. 14.6%, *p* = 0.03). The groups did not differ from each other in terms of survival from decompensation of HF (18.3% vs. 36.6%, *p* = 0.70) and survival from death (1.7% vs. 4.9%, *p* = 0.16). At the one-year follow-up, the baseline LVEF did not affect the survival rate from death or HF decompensation among patients with a progression of TR after CIED implantation. In this study, a progression by one grade was more common in group 1, but the occurrence of severe/massive TR after implantation was more specific for group 2.

## 1. Introduction

The prevalence of tricuspid regurgitation (TR) has been estimated at 85.7% of patients referred for echocardiographic evaluation [1,2]. Lead-related tricuspid regurgitation (LRTR) appears to be an important complication in patients with cardiac implantable electronic devices (CIED) [3,4,5,6]. The frequency of significant TR development after CIED implantation is not precisely determined and varies from 7.2% to 44.7% [3,4,7,8,9,10,11,12,13,14,15,16,17,18,19,20,21,22,23,24,25,26,27,28,29,30] depending on the criteria used. CIEDs may directly affect the function and structure of the tricuspid valve [3,7,8,12,14,24,29,31,32,33,34,35]. One of the most often-reported LRTR mechanisms is a leaflet impingement by a lead or the limitation of a leaflet movement caused by the adherence lead [15,24,29,31,32,33,34,36]. Other mechanisms include a leaflet perforation [12,34], damage of the subvalvular apparatus, entanglement or rupture of the chordae tendinae, papillary muscle perforation [32,33,34], and pacing-induced right ventricular dysfunction [8,37,38]. Among other factors that can cause development of the tricuspid regurgitation after CIED implantation are also mentioned a dilation of the right ventricle [9,12,15,20,24,35], an impairment of the left ventricular systolic and diastolic function [35,37,39], an increased right atrial area [9,29], an elevated right atrial pressure [14,15], and elevated pulmonary systolic pressure [9,12,40]. Other risk factors include age over 68 years [4,9,12,20], female sex [20], atrial fibrillation [9,20,32,41,42], history of mitral dysfunction [9,12,20], and an increased left atrial area [9,20,42]. Critical for LRTR development is the technique of CIED implantation. According to some researchers, a “prolapsing” technique may reduce the risk of perforation and laceration of the tricuspid valve leaflets and the subvalvular apparatus [35,39]. Rajappan suggests that “direct crossing” results in decreased risk of damage to the tricuspid valve apparatus [43]. Furthermore, the position of the lead in the center of the tricuspid orifice [7,24,31,44] or in the commissures prevents restriction of leaflet motion [7,23,24,29,31,45]. Some authors suggest that the septal and the posterior leaflets are the most affected by the leads [7,14,15,23,24,29,31,34]. As TR is associated with increased mortality [1,4,11,12,30,32] and the development of right ventricle (RV) failure [10,12,15,18,30,37], the aim of that study was a determination of TR progression after CIED implantation in patients with preserved-, mildly-, and severely reduced ejection fraction (EF), as well as tracking freedom from heart failure (HF) exacerbation and death-free survival among patients with LRTR, depending on left ventricle function, in a one-year observation.

## 2. Materials and Methods

### 2.1. Design of This Study

The prospective study involved 101 consecutive patients who received a CIED–a pacemaker (PM), implantable cardioverter–defibrillator (ICD), cardiac resynchronization therapy defibrillator (CRT-D), or cardiac resynchronization therapy pacemaker (CRT-P)–at our center between March 2020 and October 2021. The implantation procedure and baseline clinical data on preimplantation variables were retrieved from electronic medical records. Echocardiographic evaluation was performed directly before and one day after a CIED implantation and then one year after the procedure. Based on the baseline EF, the patients were divided into group 1, with preserved and mildly reduced ejection fraction (LVEF ≥ 40%) (*n* = 60), and group 2, with severe reduction of ejection fraction (LVEF < 40%) (*n* = 41). LRTR was defined as an increase of TR severity by at least one grade. The position of the lead was evaluated on the basis of X-ray scans obtained after implantation using Yu et al.’s identification criteria [36]. Follow-up data were collected during standard CIED controls on one day and on average 13 (12–16) months after implantation. Information on mortality and hospitalizations for any reason was obtained from hospital records or medical interviews.

### 2.2. Echocardiographic Examination

All patients were subjected to standard two-dimensional echocardiography assessment (Vivid 7, GE Healthcare, Chicago, IL, USA). The baseline echocardiographic characteristics were evaluated using published guidelines from the European Society of Cardiology [46,47] and included: TR grade (trivial/none 0, mild 1, moderate 2, severe 3, massive 4); right ventricular (RV) dilation; RV systolic function by RV fractional area change and tricuspid annular plane systolic excursion (TAPSE); right atrial pressure estimated on the basis of the inferior vena cava size and collapse; pulmonary artery systolic pressure as a sum of RA pressure and the peak gradient on a continuous wave Doppler of the TR; left ventricular dilation; left ventricular function by Simpson’s method; aortic stenosis grade by peak/mean gradient and valve area; aortic regurgitation by visual estimation; mitral stenosis by mean gradient and valve area; mitral regurgitation by visual estimation; and the presence of mitral or aortic valve repair or replacement.

### 2.3. Data Analysis

Data distribution was evaluated by the Kolmogorov–Smirnov test. The results were presented as a standard deviation and mean value or median and percentile distribution. Student’s *t*-test and paired Student’s *t*-test were used, respectively, for independent and dependent variables with the normal distribution. Yates’s c2 test and Fisher’s exact test were used for assessing differences among categorical parameters. The Mann–Whitney U test was used for independent nonparametric variables. The Wilcoxon test was used to compare dependent nonparametric variables. A value of *p* < 0.05 was considered statistically significant. Heart failure (HF) decompensation-free survival, based on hospitalization due to exacerbation of HF or necessity of intensification of HF treatment, and death-free survival were analyzed using the Kapplan–Meier estimator and log-rank tests. Analysis was conducted by using Statistica 10 software (TIBCO Software Inc., Palo Alto, CA, USA).

## 3. Results

### 3.1. Clinical Characteristics of Patients

The baseline characteristics of the patients are listed in Table 1. The two groups did not differ in terms of basic clinical characteristics, except for age (*p* = 0.001) and NYHA class (*p* = 0.001). Group 1 involved mainly patients with pacemakers (93% of the patients); the rest of the group constituted four patients with ICDs (two patients with hypertrophic cardiomyopathy, one with a history of ventricular fibrillation, one with a history of sustained ventricular tachycardia). In group 2, only one patient had a PM; 58.5% of the patients were equipped with ICDs and 39.1% with CRT devices.

### 3.2. Baseline Echocardiographic Characteristics

Before implantation, there were significant differences between groups in terms of the left ventricle in diastole (group 1: 93.5 (65.5–119.0) mL vs. group 2: 169.5 (154.0–219.0) mL, *p* = 0.001) and systole (group 1: 38.0 (30.0–49.0) mL vs. group 2: 124.5 (106.7–152.5) mL, *p* = 0.001), TAPSE (group 1: 21.5 (18.0–25.5) mm vs. group 2: 18.2 (18.0–20.0) mm, *p* = 0.001), and aortic stenosis and mitral regurgitation prevalence (*p* = 0.045 and *p* = 0.009, respectively). In terms of tricuspid valve function, the two groups did not differ from each other. Echocardiographic findings for both groups are listed in Table 2.

### 3.3. CIED Characteristics

The basic characteristics of the CIEDs are presented in Table 3. Pacemakers were implanted more often in group 1 (93.3% vs. 2.4%, *p* = 0.001), with implantable cardioverter–defibrillators (ICDs) and cardiac resynchronization therapy defibrillators/pacemakers (CRT-D/CRT-P) more common in group 2 (ICD: 6.7% vs. 58.5%, *p* = 0.001, and CRT-D/CRT-P: 0.0% vs. 39.1%, *p* = 0.001). Dual-chamber stimulation amounted to 80.0% in group 1 and 26.8% in group 2 (*p* = 0.001).

### 3.4. Postimplant Echocardiographic Characteristics

The mean follow-up period was 13 months (14–16 m) and was similar for both groups (*p* = 0.074). In a whole study group, TR progression by one grade was present in 34.6% of patients and by two or more grades was present in 15.8%, which is consistent with previous studies [9,10,11,13,14,16,17,18,24,26,28,30]. Similar to the preimplant data, LV end-systolic and LV end-diastolic volumes were significantly higher in group 2 (LVEDV: 104.0 (74.0–117.0) mL vs. 166.0 (119.0–211.0) ml; LV ESV 42.0 (28.9–59.0) mL vs. 106.5 (69.0–156.0) mL, group 1 vs. group 2, respectively, *p* = 0.001). TAPSE was significantly higher in group 1 (20.8 (18.0–23.0) mm vs. group 2: 17.5 (14.0–19.5) mm, *p* = 0.001). Statistically significant differences in echocardiographic parameters after CIED implantation between group 1 and 2, the same as before the implantation, concerned occurrence of the mild aortic stenosis and severe mitral regurgitation after CIED implantation and were similar in both groups (respectively, *p* = 0.235 and *p* = 1.0); mild aortic regurgitation was more frequent in group 2 (19.5%, *p* = 0.045). Incidence of particular grades of tricuspid regurgitation in both groups was similar and is presented in Table 3 (none/trivial TR, *p* = 0.825; mild TR, *p* = 0.225; moderate TR, *p* = 0.595; severe TR, *p* = 0.308; massive TR, *p* = 0.512). However, in group 1, the progression of TR by one grade occurred more often than in group 2 (43.3% vs. 21.9%, *p* = 0.033) (Table 4).

The TR degree change in both groups (Table 5) after implantation was as follows: none/trivial TR group 1 (61.7% vs. 28.3%, *p* = 0.001) and group 2 (53.6% vs. 31.7%, *p* = 0.0734); mild TR group 1 (28.3% vs. 46.7%, *p* = 0.059) and group 2 (36.6% vs. 34.1%, *p* = 1.00); moderate TR group 1 (8.3% vs. 15.0%, *p* = 0.394) and group 2 (9.7% vs. 19.5%, *p* = 0.349); severe/massive TR group 1 (1.7% vs. 10.0%, *p* = 0.114) and group 2 (0.0% vs. 14.6%, *p* = 0.026). Interestingly, the LVEF increased significantly in group 2 after CEID implantation (29.0 (21.0–32.0)% vs. 30.0 (26.0–38.0)%, *p* = 0.002), and diastolic volume reduced post implantation (169.5 (154.0–219.0) mL vs. 166.0 (119.0–211.0) mL, *p* = 0.04) (Table 5).

### 3.5. Primary Outcome Analysis (All-Cause Mortality and Hospitalizations)

**Figure 1:**
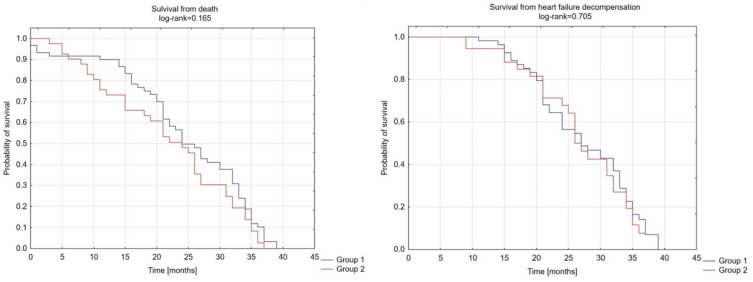
Survival of death or HF decompensation.

The two groups did not differ from each other in terms of survival of decompensation of heart failure referred to hospitalization due to decompensation of HF or intensification of HF treatment (diuretics) (group 1: 18.3% vs. group 2: 36.6%, *p* = 0.705) and survival of death (group 1: 1.7% vs. group 2: 4.9%, *p* = 0.165) (Figure 1).

The frequency of HF decompensation was almost statistically significant in patients from group 2 (group 1: 18.3% vs. 36.6%, *p* = 0.062) (Table 6).

**Figure 2:**
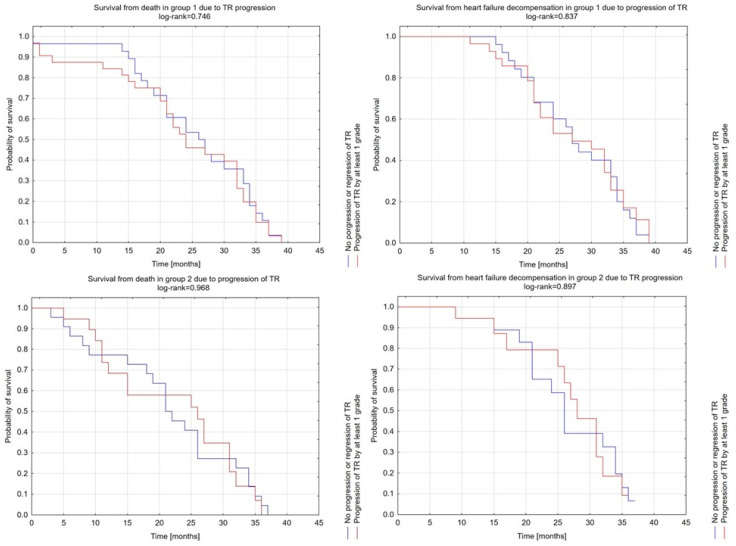
Survival from death and HF decompensation among both groups due to progression of TR.

What is more, among patients from both groups, the level of progression of TR (no progression or regression of TR vs. progression of TR by at least one grade) was irrelevant in terms of survival of hospitalization/intensification of HF treatment (group 1: *p* = 0.837 and group 2: *p* = 0.897) or death (group 1: *p* = 0.746 and group 2: *p* = 0.968) (Figure 2).

## 4. Discussion

To our knowledge, this is the first study analyzing TR progression soon (up to 12 months) after CIED implantation in patients with preserved/mildly reduced and severely reduced LVEF. Reports concerning that problem in patients with normal LV function estimate TR progression at 7.2–37.6% of cases, or 9.8–25.6% of cases if it is defined as TR worsening by two grades. If TR progression is evaluated in the general study population, without division on the basis EF, its occurrence is estimated at 7.5–44.7% of cases, or 10–38% of cases after limitation to worsening TR by two grades. The wide percentage range of presented results may be an effect of the use of varied criteria of TR progression and evaluation, as well as different tools and echo machine providers. The duration of the follow-up also varied among authors and ranged from 3 months to 139 months, which could have a significant impact on TR occurrence [4].

### 4.1. TR Progression

The general progression of TR by one grade in our study was 34.6% and by two or more grades, 15.8%, which is consistent to previous studies [3,4,7,8,9,10,11,12,13,14,15,16,17,18,19,20,21,22,23,24,25,26,27,28,29,30]. The progression of TR in group 1 was an effect of TR progression by one grade (43.3% vs. 21.9%, *p* = 0.033), which was mostly gained by the change from none/trace to mild (31.7%) (Table 4). It shows that the progression of TR in patients with preserved/mildly reduced LVEF was not hemodynamically significant. In the general study population, an increase in percentage of severe and massive TR was significant (before implantation: 0.9% vs. after implantation: 11.8%, *p* = 0.002) and concerned patients in group 2 (0.0% vs. 14.6%, *p* = 0.026). The explanation of this finding might be the almost significant difference between group 1 and group 2 in terms of progression of TR by two or more grades (10.0% vs. 24.4%, *p* = 0.093) (Table 4). An important factor is also a severe mitral insufficiency, which may be an only reason of the secondary tricuspid regurgitation. In this study, before CIED implantation, the severe mitral regurgitation was present only in group 2; however without differences in a burden of tricuspid regurgitation between the patients of both groups (none/trace TR *p* = 0.537; mild TR *p* = 0.393; medium TR *p* = 1.0 severe TR *p* = 1; massive TR = 1.0). After CIED implantation, in a group 2, a reduction of mitral regurgitation was observed, as well as an improvement in LV function and volume reduction, resulting from the restoration of intraventricular synchrony and positive LV remodeling. A number of patients with severe mitral regurgitation was finally the same in both groups, but only in the group 2 severe and massive TR occurred, probably induced by sustained RV dysfunction.

### 4.2. LV and RV Function

During follow–up, statistically significant changes in the progression of TR were observed, without any significant differences in RV function or dimensions of RA, RV, and TV diameter. There was also no progression in both groups of LV dysfunction defined as LV EF decrease or increase in LV EDV. On the contrary, LV function and LV EDV improved in group 2, probably due to CRT-D/CRT-P implantation in some of the patients. Some studies, with a similar period of observation, have demonstrated a development of the right ventricle dysfunction in patients with LRTR, unlike the patients without significant valve disease [12,29,30,42], as LRTR led to the remodeling of the right chambers of the heart [10,14,15,30]. Riesenhuber et al. and Delling et al. stated that patients with prior dilation of the RV have an increased risk of TR progression [12,20]. Papageorgiou et al. reported that in their study, new RV dysfunction occurred in 59 of 304 patients after CIED implantation [18]. Orban et al. reported that new RV dysfunction was detected in almost 20% of patients [45]. Addetia et al. suggested that in patients with underlying LV dysfunction, over time, TR will develop even in the absence of a CIED, as an effect secondary to RV enlargement and tricuspid annular dilation [29]. In our study, in both groups the dimension of RV and TV diameter was similar before implantation of CIED and did not change significantly during follow-up. The only difference between groups in terms of RV function was TAPSE, which was higher in group 1 (before implantation: 21.0 (18.0–25.5) mm vs. 18.0 (15.0–20.0) mm, *p* = 0.001, and after implantation: 21.0 (18.0–23.0) mm vs. 17.50 (14.0–19.5) mm, *p* = 0.001), which is probably due to the existence of significant LV dysfunction among patients from group 2. This particular difference between this result and previous reports is probably due to the short period of the follow-up and the small group of patients. Lee et al. determined that in some subgroups of patients, CIED implantation improved TV function and RV hemodynamic [9], which was also observed in the present study.

### 4.3. CIED Type

Both groups vary in terms of CIED type, due to different indications for its implantation, which could suggest differences in TR occurrence between them. Notwithstanding, in this study, there was no difference in TR occurrence between the groups, which is consistent with other authors [7,8,9,10,11,21,30]. Yu et al. observed that when the pacing lead is located in the RV apex, it is more likely to affect the posterior leaflet [36], and some authors suggest that the septal and posterior leaflets are the most affected by the leads, despite the pacing lead localization [7,14,15,23,24,29,31,34]. However, contrary to this, Cheng et al. [31] found that a significant increase in the PISA radius of TR after implantation of CIED was observed more often in the group with lead tips in the IVS. Their study showed that when the lead tip is in the IVS, the lead is more likely to adhere to the leaflet and chordate. What is more, various authors suggested that when the lead is in the center of the tricuspid orifice [7,24,31,44], or in one of the commissures, it prevents restriction of a leaflet motion [7,23,24,29,31,45]. Therefore, it is possible that the CIED type and its pacing leads are not the risk factor of the progression of TR after CIED implantation, because the most important aspect is the position of the lead in the TV apparatus. This reasoning may also explain why there was a greater percentage of patients with severe TR after implantation of CIED in group 2, because in group 2 the lead tip was mostly located in the RV apex (Table 4).

### 4.4. All-Cause Mortality and Hospitalizations

Papageorgiou et al. reported that moderate or severe TR and RV impairment after CIED implantation was associated with a significantly worse survival rate than in patients without them [18]. Other studies have also shown that at least moderate TR is associated with increased all-cause mortality as well as re-hospitalizations due to HF [6,10,11,12,15,19,21,30,48,49,50,51]. Delling et al. observed that the presence of a PM lead does not increase mortality risk, if it is not related to moderate or severe TR [12]. According to Offen et al., in patients with moderate or severe TR before CIED implantation, the presence of an RV lead did not increase mortality risk; however, the risk of all-cause mortality was nearly doubled for patients with moderate or severe LRTR and a median age under 77 years [49]. Y. Seo et al. reported that patients with TR not related to lead presence and HF had better responses to HF treatment than patients with LRTR in cases of hospitalization [15]. In our study, statistically significant differences in HF decompensation-free and death-free survival did not occur between groups with preserved/mildly reduced and severely reduced ejection fraction. It may be the effect of the relatively short one-year period of follow-up, as well as improvement of LV function, which occurred in patients with advanced HF within that time, and, what is all the more important in that context, that decisions concerning ICD or CRT-D implantation are given only to patients who are expected to survive more than one year in good condition. Zhang et al. in their meta-analysis showed that pacemaker implantation time was a risk factor for TR deterioration [4]. We observed an almost significant difference in the frequency of HF decompensation, based on hospitalization due to decompensation of HF or intensification of HF treatment (diuretics) in group 2 (*p* = 0.062) (Table 6).

## 5. Conclusions

A significant increase in all-cause mortality and readmissions due to HF symptoms among patients with newly moderate or severe TR, or progression of pre-existing tricuspid valve disease, remains an important clinical problem. It is possible that at a one-year follow-up, the baseline LVEF does not affect the survival rate related to death or HF decompensation among patients with a progression of TR after CIED implantation. TR progression concerns about 50% of patients after CIED implantation (in this study, 53.3% of patients with preserved/mildly reduced ejection fraction and 46.3% of patients with strongly reduced ejection fraction). It is more pronounced in patients with a higher EF as an effect of the change from a lack of TV disease to trivial/mild TR, although in patients with advanced heart failure, it is more essential as it is related with significantly more frequent occurrence of severe TV disease. Therefore, identification of the groups of patients most vulnerable to consequences of TR progression is essential for better prevention and treatment.

## Figures and Tables

**Table 1:** Patient characteristics.

	All (*n* = 101)	Group 1 (*n* = 60)	Group 2 (*n* = 41)	*p*
Men *n* (%)	57 (56.4%)	29 (48.3%)	28 (68.3%)	0.065
Age [years]	69.0 (60.0–76.0)	73 (63–78)	62 (59–72)	0.001
Weight [kg]	84.0 (74.0–94.0)	83.5 (74.0–90.0)	87.0 (74.0–94.0)	0.486
High [m]	1.70 (1.64–1.76)	1.70 (1.62–1.75)	1.74 (1.69–1.76)	0.083
Coronary artery disease *n* (%)	52 (51.5%)	28 (46.7%)	24 (58.5%)	0.311
Diabetes mellitus *n* (%)	29 (28.7%)	14 (23.3%)	15 (36.6%)	0.181
Pulmonary disease *n* (%)	7 (6.9%)	5 (8.3%)	2 (4.9%)	0.697
Atrial fibrillation *n* (%)	37 (36.6%)	20 (33.3%)	17 (41.6%)	0.528
NYHA *n* (%)				
I	58 (58.42%)	54 (90%)	4 (9.8%)	0.001
II	34 (33.66%)	6 (10%)	28 (68.3%)	0.001
III	5 (4.95%)	0 (0.0%)	5 (12.2%)	0.009
IV	3 (2.97%)	0 (0.0%)	3 (7.3%)	0.064
Bilirubin [µmol/L]	10.10 (7.2–14.7)	9.65 (7.2–14.2)	10.20 (8.9–15.3)	0.359
INR	1.07 (0.99–1.18)	1.05 (0.99–1.14)	1.09 (1.0–1.47)	0.225
Creatinine [µmol/L]	85.0 (75.0–103)	83.5 (71.0–98)	93.0 (78.0–110)	0.076
Time since CIED implantation [months]	13.0 (12.0–16.0)	13.5 (12.0–16.0)	13.0 (12.0–15.0)	0.074

CIED—cardiac implantable electronic device.

**Table 2:** Results of echocardiographic examination before CIED implantation.

	All (*n* = 101)	Group 1 (*n* = 60)	Group 2 (*n* = 41)	*p*
RV dimension in four chamber view [mm]	37.0 (35.0–40.5)	38.0 (35.0–41.0)	37.0 (33.0–40.0)	0.466
Area of RA in diastole [cm^2^]	17.30 (15.0–21.4)	18.8 (15.2–21.4)	16.4 (14.6 Q75–22.8	0.326
Area of RA in systole [cm^2^]	12.0 (10.4–16.3)	12.25 (10.8–16.3)	11.9 (10.01–16.3)	0.672
TV diameter [mm]	32.0 (29.0–38.0)	32.0 (29.0–38.0)	32.0 (29.0–38.0)	0.771
FAC [%]	38.39 (±10.65)	38.79 (±10.79)	37.91 (±10.65)	0.747
TAPSE [mm]	20.0 (17.0–23.0)	21.0 (18.0–25.5)	18.0 (15.0–20.0)	0.001
RVSP [mmHg]	33.57 (±15.82)	32.89 (±12.49)	34.38 (±19.35)	0.738
TAPSE/TRPG [mm/mmHg]	0.51 (0.43–0.9)	0.61 (0.47–0.97)	0.45 (0.27–0.87)	0.061
LV EDV [mL]	127.5 (86.0–169.0)	93.50 (65.5–119.0)	169.5 (154.0–219.0)	0.001
LV ESV [mL]	87.1 (36.0–120.0)	38.0 (30.0–49.0)	124.5 (106.75–152.5)	0.001
LVEF [%]	50.0 (30.0–55.0)	55.0 (50.0–60.0)	29.0 (21.0–32.0)	0.001
TR *n* (%) - None/trace - Mild - Medium - Severe - Massive				
	59 (58.4%)	37 (61.7%)	22 (53.6%)	0.537
	32 (31.7%)	17 (28.3%)	15 (36.6%)	0.393
	9 (8.9%)	5 (8.3%)	4 (9.7%)	1.000
	1 (0.9%)	1 (1.7%)	0 (0.0%)	1.000
	0 (0.0%)	0 (0.0%)	0 (0.0%)	1.000
Aortic stenosis *n* (%) - Mild - Medium - Severe				
	10 (9.9%)	9 (15.0%)	1 (2.4%)	0.045
	3 (2.9%)	2 (3.3%)	1 (2.4%)	1.000
	1 (0.99%)	1 (1.67%)	0 (0.0%)	1.000
Aortic regurgitation *n* (%) - Mild - Medium - Severe				
			
	11 (10.9%)	6 (10.0%)	5 (12.2%)	0.753
	3 (2.9%)	1 (1.7%)	2 (4.9%)	0.564
	0 (0.0%)	0 (0.0%)	0 (0.0%)	1.000
Mitral stenosis *n* (%) - Mild - Medium - Severe				
	0 (0.0%)	0 (0.0%)	0 (0.0%)	1.000
	0 (0.0%)	0 (0.0%)	0 (0.0%)	1.000
	0 (0.0%)	0 (0.0%)	0 (0.0%)	1.000
Mitral regurgitation *n* (%) - Mild - Medium - Severe				
			
	30 (29.7%)	19 (31.7%)	11 (26.8%)	0.661
	9 (8.9%)	3 (5.0%)	6 (14.6%)	0.153
	5 (4.9%)	0 (0.0%)	5 (12.2%)	0.009

EDV—end-diastolic volume, ESV—end-systolic volume, FAC—fractional area change, LVEF—left ventricle ejection fraction, RA—right atrium, RV—right ventricle, TAPSE—tricuspid annular plane systolic excursion, TAPSE/TRPG—tricuspid annular plane systolic excursion/tricuspid regurgitation peak gradient, TV—tricuspid valve.

**Table 3:** Results of CIED controls and echocardiographic examinations after one year of follow-up.

	All (*n* = 101)	Group 1 (*n* = 60)	Group 2 (*n* = 41)	*p*
CIED type and parameters				
Type of device *n*(%)				
- PPM - ICD - CRT-P/CRT-D	57 (56.4%)	56 (93.3%)	1 (2.4%)	0.001
	28 (27.7%)	4 (6.7%)	24 (58.5%)	0.001
	16 (15.8%)	0 (0.0%)	16 (39.1%)	0.001
Pacing mode *n*(%) - AAI - VVI - DDD - BiV				
	0 (0.0%)	0 (0.0%)	0 (0.0%)	1.000
	27 (26.7%)	12 (20.0%)	15 (36.6%)	0.0721
	59 (58.4%)	48 (80.0%)	11 (26.8%)	0.001
	15 (14.8%)	0 (0.0%)	15 (36.6%)	0.001
Percentage of ventricular pacing [%]	19.3 (1.0–93.0)	23.0 (1.5–90.0)	4.0 (1.0–97.5)	0.460
Echocardiographic parameters				
TV diameter [mm]	33.0 (30.0–38.0)	32.0 (29.0–37.0)	33.0 (31.0–38.0)	0.496
FAC [%]	41.74 (±11.17)	43.05 (±11.57)	39.76 (±10.39)	0.172
TAPSE [mm]	19.0 (16.0–23.0)	21.0 (18.0–23.0)	17.50 (14.0–19.5)	0.001
RVSP [mmHg]	30.0 (±14.2)	29.76 (±14.5)	30.39 (±13.9)	0.853
TAPSE/TRPG [mm/mmHg]	0.62 (0.46–0.94)	0.74 (0.5–1.0)	0.49 (0.43–0.82)	0.077
LV EDV [mL]	115.6 (86.0–166.0)	104.0 (74.0–117.0)	166.0 (119.0–211.0)	0.001
LV ESV [mL]	59.0 (38.5–104.5)	42.0 (28.9–59.0)	106.50 (69.0–156.0)	0.001
LVEF [%]	49.0 (31.0–58.0)	56.0 (50.0–60.0)	30.0 (26.0–38.0)	0.001
TR *n* (%) - None/trace - Mild - Medium - Severe - Massive				
	30 (29.7%)	17 (28.3%)	13 (31.7%)	0.825
	42 (41.6%)	28 (46.7%)	14 (34.1%)	0.225
	17 (16.8%)	9 (15.0%)	8 (19.5%)	0.595
	10 (9.9%)	4 (6.7%)	6 (14.6%)	0.308
	2 (1.9%)	2 (3.3%)	0 (0.0%)	0.512
Aortic stenosis *n* (%) - Mild - Medium - Severe				
	7 (6.3%)	6 (10.0%)	1 (2.4%)	0.235
	3 (2.9%)	1 (1.7%)	2 (4.9%)	0.564
	1 (0.9%)	1 (1.7%)	0 (0.0%)	1.000
Aortic regurgitation *n* (%) - Mild - Medium - Severe				
			
	11 (10.9%)	3 (5.0%)	8 (19.5%)	0.046
	3 (2.9%)	1 (1.7%)	2 (4.9%)	0.564
	0 (0.0%)	0 (0.0%)	0 (0.0%)	1.000
Mitral stenosis *n* (%) - Mild - Medium - Severe				
	4 (3.9%)	4 (6.7%)	0 (0.0%)	0.1442
	1 (0.9%)	1 (1.7%)	0 (0.0%)	1.000
	0 (0.0%)	0 (0.0%)	0 (0.0%)	1.000
Mitral regurgitation *n* (%) - Mild - Medium - Severe				
			
	29 (28.7%)	16 (26.7%)	13 (31.7%)	0.656
	16 (15.8%)	7 (11.7%)	9 (21.9%)	0.178
	2 (1.9%)	1 (1.7%)	1 (2.4%)	1.000

AAI—single atrial stimulation, BiV—biventricular stimulation, CIED—cardiac implantable electronic device, CRT-D/CRT-P—cardiac resynchronization therapy defibrillator/cardiac resynchronization therapy pacemaker, DDD—dual chamber stimulation, EDV—end-diastolic volume, ESV—end-systolic volume, FAC—fractional area change, ICD—implantable cardioverter–defibrillator, LV—left ventricle, LVEF—left ventricle ejection fraction, PM—pacemaker, RVSP—right ventricular systolic pressure, TAPSE—tricuspid annular plane systolic excursion, TAPSE/TRPG—tricuspid annular plane systolic excursion/tricuspid regurgitation peak gradient, TR—tricuspid regurgitation, TV—tricuspid valve, VVI—single ventricle stimulation.

**Table 4:** Dynamic of progression of TR after CIED implantation and position of the lead.

	All (*n* = 101)	Group 1 (*n* = 60)	Group 2 (*n* = 41)	*p*
PROGRESSION OF TR				
No progression *n*(%)	41 (40.6%)	24 (40.0%)	17 (41.46%)	0.522
TR progression by 1 grade *n* (%)	35 (34.6%)	26 (43.3%)	9 (21.9%)	0.033
- None/trace to mild - Mild to moderate - Moderate to severe - Severe to massive	25 (24.75%)	19 (31.67%)	6 (14.63%)	0.0179
	5 (4.95%)	3 (5.0%)	2 (4.88%)	1.000
	4 (3.96%)	3 (5.0%)	1 (2.44%)	0.6445
	1 (0.99%)	1 (1.67%)	0 (0.0%)	1.000
TR progression by ≥2 grades *n* (%)	16 (15.8%)	6 (10.0%)	10 (24.4%)	0.093
- None/trace to medium - None/trace to severe - Mild to severe - Moderate do massive	9 (8.91%)	4 (6.67%)	5 (12.19%)	0.4796
	2 (1.98%)	1 (1.67%)	1 (2.44%)	1.0000
	5 (4.95%)	1 (1.67%)	4 (9.76%)	0.1551
	0 (0.0%)	0 (0.0%)	0 (0.0%)	1.0000
Regression *n*(%)	9 (8.9%)	4 (6.67%)	5 (12.19%)	0.479
POSITION OF THE LEAD				
RVA *n*(%)	24 (23.76%)	7 (11.67%)	17 (41.46%)	0.0008
Non-RVA *n*(%)	77 (76.24%)	53 (88.33%)	24 (58.53%)	0.0008

Non-RVA—non-right ventricle apex position, RVA—right ventricle apex position, TR—tricuspid regurgitation.

**Table 5:** Echocardiographic parameter changes after implantation of CIED.

Parameter	All (*n* = None/trace to severe101)			Group 1 (*n* = 60)			Group 2 (*n* = 41)		
	Before Implantation	After Implantation	*p*	Before Implantation	After Implantation	*p*	Before Implantation	After Implantation	*p*
TR grade									
*n* (%)									
None	59 (58.4%)	30 (29.7%)	0.001	37 (61.7%)	17 (28.3%)	0.01	22 (53.6%)	13 (31.7%)	0.073
Mild	32 (31.7%)	42 (41.6%)	0.189	17 (28.3%)	28 (46.7%)	0.06	15 (36.6%)	14 (34.1%)	1.000
Medium	9 (8.9%)	17 (16.8%)	0.140	5 (8.3%)	9 (15.0%)	0.39	4 (9.7%)	8 (19.5%)	0.349
≥Severe	1 (0.9%)	12 (11.8%)	0.002	1 (1.7%)	6 (10.0%)	0.11	0 (0.0%)	6 (14.6%)	0.026
RA in diastole [cm^2^]	17.3 (15.0–21.4)	19.3 (16.2–23.2)	0.150	18.8 (15.2–21.4)	19.9 (16.6–23.5)	0.127	16.4 (14.6–22.8)	18.6 (15.4–23.2)	0.628
RA in systole [cm^2^]	12.0 (10.4–16.3)	13.0 (11.3–16.9)	0.330	12.3 (10.8–16.3)	13.0 (11.3–16.9)	0.598	11.9 (10.0–16.3)	13.2 (11.2–16.9)	0.425
TV diameter [mm]	32.0 (29.0–38.0)	33.0 (30.0–38.0)	0.385	32.0 (29.0–38.0)	32.0 (29.0–37.0)	0.635	32.0 (29.0–38.0)	33.0 (31.0–38.0)	0.342
RV in 4 chambers [mm]	37.0 (35.0–40.5)	38.0 (36.0–42.0)	0.163	38.0 (35.0–41.0)	38.0 (36.0–40.0)	0.898	37.0 (33.0–40.0)	38.0 (35.0–44.0)	0.103
RVSP [mmHg]	33.6 (±15.82)	30.0 (±14.2)	0.185	32.9 (±12.5)	29.7(±14.5)	0.339	34.4 (±19.4)	30.4 (±13.9)	0.388
FAC RV [%]	38.4 (±10.6)	41.7 (±11.2)	0.063	38.8 (±10.8)	43.0 (±11.6)	0.084	37.9 (±10.6)	39.7 (±10.4)	0.483
TAPSE [mm]	20.0 (17.0–23.0)	19.0 (16.0–23.0)	0.318	21.0 (18.0–25.5)	21.0 (18.0–23.0)	0.488	18.0 (15.0–20.0)	17.5 (14.0–19.5)	0.405
TAPSE/TRPG [mm/mmHg]	0.51 (0.4–0.9)	0.62 (0.5–0.9)	0.125	0.6 (0.5–0.9)	0.7 (0.5–1.0)	0.277	0.4 (0.3–0.9)	0.5 (0.4–0.8)	0.325
LV EDV [mL]	127.5 (86.0–169.0)	115.6 (86.0–166.0)	0.383	93.5 (65.5–119.0)	104.0 (74.0–117.0)	0.293	169.5 (154.0–219.0)	166.0 (119.0–211.0)	0.038
LV ESV [mL]	87.1 (36.0–120.0)	59.0 (38.5–104.5)	0.355	38.0 (30.0–49.0)	42.0 (28.9–59.0)	0.325	124.5 (106.8–152.5)	106.5 (69.0–156.0)	0.091
LV EF [%]	50.0 (30.0–55.0)	49.0 (31.0–58.0)	0.005	55.0 (50.0–60.0)	56.0 (50.0–60.0)	0.288	29.0 (21.0–32.0)	30.0 (26.0–38.0)	0.002

CIED—cardiac implantable electronic device, EDV—end-diastolic volume, ESV—end-systolic volume, FAC—fractional area change, LV EF—left ventricle ejection fraction, TAPSE—tricuspid annular plane systolic excursion, TAPSE/TRPG—tricuspid annular plane systolic excursion/tricuspid regurgitation peak gradient, TR—tricuspid regurgitation, TV—tricuspid valve.

**Table 6:** Occurrence of death due to any reason or heart failure decompensation (hospitalization due to decompensation of HF/intensification of HF treatment (diuretics)).

	All (*n* = 101)	Group 1 (*n* = 60)	Group 2 (*n* = 41)	*p*
Death due to any reason *n* (%)	3 (2.97%)	1 (1.67%)	2 (4.87%)	0.5645
HF decompensation *n* (%)	26 (25.7%)	11 (18.33%)	15 (36.6%)	0.0625

HF—heart failure.

## Data Availability

The data presented in this study are available on request from the corresponding author. The data are not publicly available due to restrictions eg privacy.

## References

[1] Di Mauro M., Bezante G.P., Di Baldassarre A. (2013). Review: Functional tricuspid regurgitation: An underestimated issue. Int. J. Cardiol..

[2] Singh J.P., Evans J.C., Levy D., Larson M.G., Freed L.A., Fuller D.L., Lehman B., Benjamin E.J. (1999). Prevalence and clinical determinants of mitral, tricuspid, and aortic regurgitation (The Framingham Heart Study). Am. J. Cardiol..

[3] Tatum R., Maynes E.J., Wood C.T., Deb A.K., Austin M.A., O’Malley T.J., Choi J.H., Massey H.T., Morris R.J., Pavri B.B. (2021). Tricuspid regurgitation associated with implantable electrical device insertion: A systematic review and meta-analysis. Pacing Clin. Electrophysiol..

[4] Zhang X.X., Wei M., Xiang R., Lu Y.M., Zhang L., Li Y.D., Zhang J.H., Xing Q., Tu-Erhong Z.K., Tang B.P. (2022). Incidence, Risk Factors, and Prognosis of Tricuspid Regurgitation After Cardiac Implantable Electronic Device Implantation: A Systematic Review and Meta-analysis. J. Cardiothorac. Vasc. Anesth..

[5] Gelves-Meza J., Lang R.M., Valderrama-Achury M.D., Zamorano J.L., Vargas-Acevedo C., Medina H.M., Salazar G. (2022). Tricuspid Regurgitation Related to Cardiac Implantable Electronic Devices: An Integrative Review. J. Am. Soc. Echocardiogr..

[6] Wang N., Fulcher J., Abeysuriya N., McGrady M., Wilcox I., Celermajer D., Lal S. (2019). Tricuspid regurgitation is associated with increased mortality independent of pulmonary pressures and right heart failure: A systematic review and meta-analysis. Eur. Heart J..

[7] Mediratta A., Addetia K., Yamat M., Moss J.D., Nayak H.M., Burke M.C., Weinert L., Maffessanti F., Jeevanandam V., Mor-Avi V. (2014). 3D Echocardiographic Location of Implantable Device Leads and Mechanism of Associated Tricuspid Regurgitation. JACC Cardiovasc. Imaging.

[8] Fanari Z., Hammami S., Hammami M.B., Hammami S., Shuraih M. (2015). The effects of right ventricular apical pacing with transvenous pacemaker and implantable cardioverter defibrillator on mitral and tricuspid regurgitation. J. Electrocardiol..

[9] Lee R.C., Friedman S.E., Kono A.T., Greenberg M.L., Palac R.T. (2015). Tricuspid Regurgitation Following Implantation of Endocardial Leads: Incidence and Predictors. PACE Pacing Clin. Electrophysiol..

[10] Arabi P., Özer N., Ateş A.H., Yorgun H., Oto A., Aytemir K. (2015). Effects of pacemaker and implantable cardioverter defibrillator electrodes on tricuspid regurgitation and right sided heart functions. Cardiol. J..

[11] Al-Bawardy R., Krishnaswamy A., Rajeswaran J., Bhargava M., Wazni O., Wilkoff B., Tuzcu E.M., Martin D., Thomas J., Blackstone E. (2015). Tricuspid regurgitation and implantable devices. PACE Pacing Clin. Electrophysiol..

[12] Delling F.N., Hassan Z.K., Piatkowski G., Tsao C.W., Rajabali A., Markson L.J., Zimetbaum P.J., Manning W.J., Chang J.D., Mukamal K.J. (2016). Tricuspid regurgitation and mortality in patients with transvenous permanent pacemaker leads. Am. J. Cardiol..

[13] Rydlewska A., Ząbek A., Boczar K., Lelakowski J., Małecka B. (2017). Tricuspid valve regurgitation in the presence of endocardial leads—An underestimated problem. Postep. Kardiol. Interwencyjnej.

[14] Nakajima H., Seo Y., Ishizu T., Iida N., Sato K., Yamamoto M., MacHino-Ohtsuka T., Nogami A., Ohte N., Ieda M. (2020). Features of lead-induced tricuspid regurgitation in patients with heart failure events after cardiac implantation of electronic devices—A three-dimensional echocardiographic study. Circ. J..

[15] Seo Y., Nakajima H., Ishizu T., Iida N., Sato K., Yamamoto M., Machino-Ohtsuka T., Nogami A., Ohte N., Ieda M. (2020). Comparison of Outcomes in Patients with Heart Failure with Versus without Lead-Induced Tricuspid Regurgitation after Cardiac Implantable Electronic Devices Implantations. Am. J. Cardiol..

[16] Seo J., Kim D.Y., Cho I., Hong G.R., Ha J.W., Shim C.Y. (2020). Prevalence, predictors, and prognosis of tricuspid regurgitation following permanent pacemaker implantation. PLoS ONE.

[17] Paniagua D., Aldrich H.R., Lieberman E.H., Lamas G.A., Agatston A.S. (1998). Increased prevalence of significant tricuspid regurgitation in patients with transvenous pacemakers leads. Am. J. Cardiol..

[18] Papageorgiou N., Falconer D., Wyeth N., Lloyd G., Pellerin D., Speechly-Dick E., Segal O.R., Lowe M., Rowland E., Lambiase P.D. (2020). Effect of tricuspid regurgitation and right ventricular dysfunction on long-term mortality in patients undergoing cardiac devices implantation: >10-year follow-up study. Int. J. Cardiol..

[19] Lee W.C., Fang H.Y., Chen H.C., Chen Y.L., Tsai T.H., Pan K.L., Lin Y.S., Liu W.H., Chen M.C. (2021). Progressive tricuspid regurgitation and elevated pressure gradient after transvenous permanent pacemaker implantation. Clin. Cardiol..

[20] Riesenhuber M., Spannbauer A., Gwechenberger M., Pezawas T., Schukro C., Stix G., Schneider M., Goliasch G., Anvari A., Wrba T. (2021). Pacemaker lead-associated tricuspid regurgitation in patients with or without pre-existing right ventricular dilatation. Clin. Res. Cardiol..

[21] Kanawati J., Ng A.C.C., Khan H., Yu C., Hyun K., Abed H., Kritharides L., Sy R.W. (2021). Long-Term Follow-Up of Mortality and Heart Failure Hospitalisation in Patients with Intracardiac Device-Related Tricuspid Regurgitation. Hear Lung Circ..

[22] Mangieri A., Montalto C., Pagnesi M., Jabbour R.J., Rodés-Cabau J., Moat N., Colombo A., Latib A. (2017). Mechanism and Implications of the Tricuspid Regurgitation: From the Pathophysiology to the Current and Future Therapeutic Options. Circ. Cardiovasc. Interv..

[23] Poorzand H., Tayyebi M., Hosseini S., Bakavoli A.H., Keihanian F., Jarahi L., Hamadanchi A. (2021). Predictors of worsening TR severity after right ventricular lead placement: Any added value by post-procedural fluoroscopy versus three–dimensional echocardiography?. Cardiovasc. Ultrasound.

[24] Seo Y., Ishizu T., Nakajima H., Sekiguchi Y., Watanabe S., Aonuma K. (2008). Clinical utility of 3-dimensional echocardiography in the evaluation of tricuspid regurgitation caused by pacemaker leads. Circ. J..

[25] Kim J.B., Spevack D.M., Tunick P.A., Bullinga J.R., Kronzon I., Chinitz L.A., Reynolds H.R. (2008). The Effect of Transvenous Pacemaker and Implantable Cardioverter Defibrillator Lead Placement on Tricuspid Valve Function: An Observational Study. J. Am. Soc. Echocardiogr..

[26] Webster G., Margossian R., Alexander M.E., Cecchin F., Triedman J.K., Walsh E.P., Berul C.I. (2008). Impact of transvenous ventricular pacing leads on tricuspid regurgitation in pediatric and congenital heart disease patients. J. Interv. Card. Electrophysiol..

[27] Klutstein M., Balkin J., Butnaru A., Ilan M., Lahad A., Rosenmann D. (2009). Tricuspid incompetence following permanent pacemaker implantation. PACE Pacing Clin. Electrophysiol..

[28] Alizadeh A., Sanati H.R., Haji-Karimi M., Yazdi A.H., Rad M.A., Haghjoo M., Emkanjoo Z. (2011). Induction and aggravation of atrioventricular valve regurgitation in the course of chronic right ventricular apical pacing. Europace.

[29] Addetia K., Maffessanti F., Mediratta A., Yamat M., Weinert L., Moss J.D., Nayak H.M., Burke M.C., Patel A.R., Kruse E. (2014). Impact of Implantable Transvenous Device Lead Location on Severity of Tricuspid Regurgitation. J. Am. Soc. Echocardiogr..

[30] Höke U., Auger D., Thijssen J., Wolterbeek R., Van Der Velde E.T., Holman E.R., Schalij M.J., Bax J.J., Delgado V., Marsan N.A. (2014). Significant lead-induced tricuspid regurgitation is associated with poor prognosis at long-term follow-up. Heart.

[31] Cheng Y., Gao H., Tang L., Li J., Yao L. (2016). Clinical utility of three-dimensional echocardiography in the evaluation of tricuspid regurgitation induced by implantable device leads. Echocardiography.

[32] Polewczyk A., Jacheć W., Nowosielecka D., Tomaszewski A., Brzozowski W., Szczęśniak-Stańczyk D., Duda K., Kutarski A. (2022). Lead dependent tricuspid valve dysfunction-risk factors, improvement after transvenous lead extraction and long-term prognosis. J. Clin. Med..

[33] Polewczyk A., Kutarski A., Tomaszewski A., Brzozowski W., Czajkowski M., Polewczyk M., Janion M. (2013). Lead dependent tricuspid dysfunction: Analysis of the mechanism and management in patients referred for transvenous lead extraction. Cardiol. J..

[34] Lin G., Nishimura R.A., Connolly H.M., Dearani J.A., Sundt T.M., Hayes D.L. (2005). Severe Symptomatic Tricuspid Valve Regurgitation Due to Permanent Pacemaker or Implantable Cardioverter-Defibrillator Leads. J. Am. Coll. Cardiol..

[35] Trankle C.R., Gertz Z.M., Koneru J.N., Kasirajan V., Nicolato P., Bhardwaj H.L., Ellenbogen K.A., Kalahasty G. (2018). Severe tricuspid regurgitation due to interactions with right ventricular permanent pacemaker or defibrillator leads. PACE Pacing Clin. Electrophysiol..

[36] Yu Y.J., Chen Y., Lau C.P., Liu Y.X., Wu M.Z., Chen Y.Y., Ho L.M., Tse H.F., Yiu K.H. (2020). Nonapical Right Ventricular Pacing Is Associated with Less Tricuspid Valve Interference and Long-Term Progress of Tricuspid Regurgitation. J. Am. Soc. Echocardiogr..

[37] Saito M., Iannaccone A., Kaye G., Negishi K., Kosmala W., Marwick T.H. (2015). Effect of Right Ventricular Pacing on Right Ventricular Mechanics and Tricuspid Regurgitation in Patients with High-Grade Atrioventricular Block and Sinus Rhythm (from the Protection of Left Ventricular Function during Right Ventricular Pacing Study). Am. J. Cardiol..

[38] Beurskens N.E.G., Tjong F.V.Y., De Bruin-Bon R.H.A., Dasselaar K.J., Kuijt W.J., Wilde A.A.M., Knops R.E. (2019). Impact of Leadless Pacemaker Therapy on Cardiac and Atrioventricular Valve Function Through 12 Months of Follow-Up. Circ. Arrhythmia Electrophysiol..

[39] Chang J.D., Manning W.J., Ebrille E., Zimetbaum P.J. (2017). Tricuspid Valve Dysfunction Following Pacemaker or Cardioverter-Defibrillator Implantation. J. Am. Coll. Cardiol..

[40] Mutlak D., Aronson D., Lessick J., Reisner S.A., Dabbah S., Agmon Y. (2009). Functional tricuspid regurgitation in patients with pulmonary hypertension: Is pulmonary artery pressure the only determinant of regurgitation severity?. Chest.

[41] Cho M.S., Kim J., Lee J.B., Nam G.B., Choi K.J., Kim Y.H. (2019). Incidence and predictors of moderate to severe tricuspid regurgitation after dual-chamber pacemaker implantation. PACE Pacing Clin. Electrophysiol..

[42] Dokainish H., Elbarasi E., Masiero S., Van de Heyning C., Brambatti M., Ghazal S., AL-Maashani S., Capucci A., Buikema L., Leong D. (2015). Prospective study of tricuspid valve regurgitation associated with permanent leads in patients undergoing cardiac rhythm device implantation: Background, rationale, and design. Glob. Cardiol. Sci. Pract..

[43] Rajappan K. (2009). Permanent pacemaker implantation technique: Part II. Heart.

[44] Addetia K., Harb S.C., Hahn R.T., Kapadia S., Lang R.M. (2019). Cardiac Implantable Electronic Device Lead-Induced Tricuspid Regurgitation. JACC Cardiovasc. Imaging.

[45] Orban M., Orban M., Hausleiter J., Braun D. (2020). Tricuspid regurgitation and right ventricular dysfunction after cardiac device implantation—Is it time for intra-procedural TEE-guided lead implantation?. Int. J. Cardiol..

[46] Lancellotti P., Tribouilloy C., Hagendorff A., Popescu B.A., Edvardsen T., Pierard L.A., Badano L., Zamorano J.L. (2013). Recommendations for the echocardiographic assessment of native valvular regurgitation: An executive summary from the European Association of Cardiovascular Imaging. Eur. Heart J. Cardiovasc. Imaging.

[47] Lang R.M., Badano L.P., Mor-Avi V., Afilalo J., Armstrong A., Ernande L., Flachskampf F.A., Foster E., Goldstein S.A., Kuznetsova T. (2015). Recommendations for cardiac chamber quantification by echocardiography in adults: An update from the American society of echocardiography and the European association of cardiovascular imaging. Eur. Heart J. Cardiovasc. Imaging.

[48] Marijon E., Trinquart L., Otmani A., Leclercq C., Fauchier L., Chevalier P., Klug D., Defaye P., Lellouche N., Mansourati J. (2010). Predictors for short-term progressive heart failure death in New York Heart Association II patients implanted with a cardioverter defibrillator-the EVADEF study. Am. Heart J..

[49] Offen S., Strange G., Playford D., Celermajer D.S., Stewart S. (2023). Prevalence and prognostic impact of tricuspid regurgitation in patients with cardiac implantable electronic devices: From the national echocardiography database of Australia. Int. J. Cardiol..

[50] Chorin E., Rozenbaum Z., Topilsky Y., Konigstein M., Ziv-Baran T., Richert E., Keren G., Banai S. (2020). Tricuspid regurgitation and long-term clinical outcomes. Eur. Heart J. Cardiovasc. Imaging.

[51] Cork D.P., Mccullough P.A., Mehta H.S., Barker C.M., Van Houten J., Gunnarsson C., Ryan M.P., Baker E.R., Mollenkopf S., Verta P. (2020). The economic impact of clinically significant tricuspid regurgitation in a large, administrative claims database. J. Med. Econ..

